# Human adults prefer to cooperate even when it is costly

**DOI:** 10.1098/rspb.2022.0128

**Published:** 2022-04-27

**Authors:** Arianna Curioni, Pavel Voinov, Matthias Allritz, Thomas Wolf, Josep Call, Günther Knoblich

**Affiliations:** ^1^ Department of Cognitive Science, Central European University, 1100 Wien, Austria; ^2^ School of Psychology and Neuroscience, University of St Andrews, St Andrews, Fife KY16 9JU, UK; ^3^ Primate Research Institute, Kyoto University, Inuyama, Aichi 484-8506, Japan

**Keywords:** utility, joint action, costs, cooperation

## Abstract

Joint actions are cooperative activities where humans coordinate their actions to achieve individual and shared goals. While the motivation to engage in joint action is clear when a goal cannot be achieved by individuals alone, we asked whether humans are motivated to act together even when acting together is not necessary and implies incurring additional costs compared to individual goal achievement. Using a utility-based empirical approach, we investigated the extent of humans' preference for joint action over individual action, when the instrumental costs of performing joint actions outweigh the benefits. The results of five experiments showed that human adults have a stable preference for joint action, even if individual action is more effective to achieve a certain goal. We propose that such preferences can be understood as ascribing additional reward value to performing actions together.

## 1. Introduction

Humans are motivated to follow opportunities for social interactions [1]. Extensive research in comparative, evolutionary and developmental psychology has shown that, by virtue of a special motivation to engage in cooperative activities with multiple partners independently of kin, humans have developed a suite of cooperative behaviours unparalleled among other animals [2–4]. In contrast with our closest evolutionary relatives who engage in cooperative activities when it is instrumental to achieve their goal [5,6], human adults are willing and able to coordinate with others to cooperate in a variety of domains, managing to establish large scale joint actions as well as mastering interpersonal coordination at the millisecond scale [7].^1^

Humans’ cooperative skills and motivation emerge early on in ontogeny. Evidence from developmental psychology shows that, beginning in their second year of life, toddlers engage in cooperative activities such as helping or complementing a partner's action to achieve a common goal [9–11]. Along with a precocious ability to cooperate with others, humans show a strong motivation to do so. Evidence indicates that from around the age of 3 years children choose to cooperate with partners even when cooperation is not necessary for achieving their desired outcome [6]. Both the psychological and evolutionary foundations of humans' motivation to engage in cooperative activities where the benefits outweigh the costs are clear: a preference for cooperation is especially valuable when it allows humans and other primates to reach outcomes that could not be achieved otherwise [8,12]. Here we investigate whether humans show a preference for cooperation even when its costs outweigh its benefit, i.e. when cooperation does not represent the most efficient means to achieve the desired goal.

Choosing between action alternatives implies a cost–benefit analysis. To make such decisions, we apply an intuitive utility calculus where we weigh the expected benefits of each option against anticipated costs [13–15]. Most often, to make such comparisons we unify ‘abstract’ units of cost and reward into a common currency; for example, when deciding what to eat at a restaurant or choosing among different routes to reach a certain destination [16]. The computation of these costs and benefits is influenced by many factors such as the risk associated with the choice, the time delay to the outcomes, and importantly for this study, social considerations [17]. In everyday situations, in line with the principle of effort avoidance, we expect human adults to minimize their effort and maximize their welfare [18,19], although their decisions may be far from complying with perfect rationality [20,21]. The performance of goal-directed actions is also guided by optimization and utility [22,23]. The selection of action plans is determined by prior knowledge of actions' costs and outcomes [22]: in fact, people decide how to act using information about relative costs of action implementations and strive towards optimality [24]. Interestingly, principles of optimality in the action domain are also foundational to humans’ ability to make sense of others' behaviour from very early on [25–27]. In developmental psychology, utility models have been recently adopted to explain how infants and children understand, compare and select individual actions in social contexts (naive utility calculus model, [28–32]). Altogether, optimality and utility principles constitute the pillars of humans’ ability to understand intentional actions and develop optimal action planning [33].

Here, we apply principles formulated for action understanding and performance in individual contexts to investigate whether agents follow utility principles when deciding whether to act alone or together. We focus on a scenario where an individual must choose between acting alone or together to perform a task. Choosing to act together may be optimal for the individual if cooperation pays off the invested costs, as it often does. However, acting together may not always be the best (less costly) means to achieve the desired outcome. Research on joint action shows that acting together requires costly cognitive computations, such as mentalizing and representing conflicting perspectives [34,35]. Individuals need to recruit specialized prediction and monitoring processes [36,37], and dedicated task and action representations [38,39]. Spatial and temporal coordination requires fine sensorimotor skills and predictive abilities [40]. Distributing tasks among partners significantly increases cognitive load [41,42]. When acting together, individuals modify their actions to facilitate the understanding of their goals. To do so, they incur significant behavioural costs such as deviation from optimal action performance [43–45]. The nature of these costs depends on the specific action performed and the kind of coordination required. For instance, when carrying a box together, an agent will incur temporal and spatial coordination costs that are not present when carrying a box alone. Costs may be high if the box is hard to grasp for one or both co-agents. The way such costs are evaluated is influenced by the expected benefits that can be achieved. From evolutionary theories on cooperation, we know that individuals derive and expect various benefits from doing things together with conspecifics—from affiliation to reputation and social status, to future reciprocation [46]. From research on joint action, we also know that individuals engage in joint actions because of the sense of commitment they feel towards cooperative partners [47]. Although not always directly relevant to the task at hand, expected social benefits can play a role in individuals' decisions towards cooperation in everyday situations.

Although much evidence indicates that doing things together can be costly, it is an open question whether costs and benefits of joint action influence the decisions to cooperate to achieve a certain goal. We propose a new approach to address whether human adults’ prefer to achieve goals together rather than alone, and if this preference depends on the costs they incur when coordinating with others.

Specifically, we investigate participants' decisions when faced with two alternative means to achieve a goal: performing individual versus joint actions. Using a utility-based logic [29], we can predict if participants have a preference for joint actions and also establish if it depends on the additional instrumental costs they incur to coordinate with a partner. This allows us to discuss the implications of a preference for joint action in supporting humans’ motivation to engage in cooperative activities.

**Figure 1.**
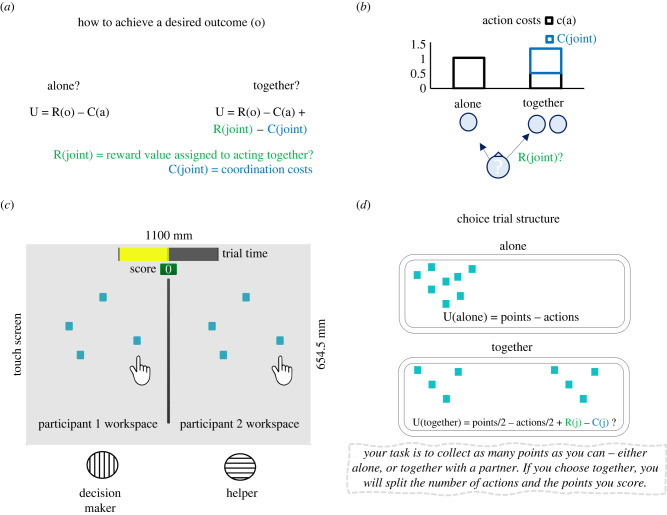
(*a*) Formalization of alone and together actions in terms of individual utility. U is the utility expected from action a to reach outcome o: difference between R(o), the subjective reward expected from outcome o of action a, and C(a), the subjective cost expected from instrumental action a. We specify the costs C(joint) for the coordination cost if the action is performed together. We specify R(joint) as the expected reward if the action a is performed together. All parameters reflect estimated (subjective) rather than objective values. (*b*) Performing the task together is equivalent to alone only if agent and partner are twice as fast at completing the task together compared to alone. Choosing together when the cost (time) of performing actions together exceeds the cost to perform actions alone is suboptimal in terms of instrumental utility. If individuals choose to act together when the costs are higher than acting alone, this indicates a preference for joint action: this preference could originate in a reward value R(j) assigned to acting together. (*c*) Task set-up. (*d*) Choice structure: when choosing together, the actions (cost) required to complete the trial are split with the partner. The number of points (reward) is halved. (Online version in colour.)

We model a scenario where a participant needs to complete a task either alone or together with a collaborative partner (figure 1*a*). We here tested whether adult participants will perform, to some degree, a cost–benefit analysis that weighs the value of the action alternatives against their costs. The action alternatives we will test in our experiments are alone versus together (Experiment (Exp.) 1, 3 and 4), and uni-manual versus bi-manual action (Exp. 2 and 5). In our scenario, when the participant chooses to complete the task alone, she incurs a given action cost C and gains a given score S. When the participant chooses to complete it together with a partner, she halves her own action costs (C/2), and her reward (score, S/2). Therefore, choosing together is suboptimal in terms of maximizing the score per trial. The alone and together modes could be equivalent from an instrumental utility standpoint only if the participant was twice as fast at solving the task together compared to alone**.** The suboptimal choice in terms of score could be compensated by performing more trials and gaining more opportunities to score points (figure 1*d*).

If the participant incurs additional costs (time/number of actions) when acting together, alone and together modes are no longer equivalent in terms of relative utility. It follows that, if the participant decides to perform the task together despite the extra cost they incur coordinating with their partner, such decision reflects a preference for joint action (figure 1*b*). Such preference in this scenario cannot be explained by instrumental utility only. With ‘instrumental utility’, we refer to the utility assigned to instrumental actions, i.e. the costs associated with performing the action necessary to achieve an outcome, and the value (reward) assigned to that outcome (score).

We implemented this scenario in a touchscreen-based game where we asked individuals to choose whether to perform a task together or alone. The participants' task was to collect as many points as possible by clearing two-dimensional items (boxes) displayed on a screen (figure 1*c*).

In a series of five experiments, we tested whether participants have a preference for performing a task together with a partner despite the higher costs that coordinating with a partner implies. We introduce a series of solo tasks where individuals need to decide whether to act uni- or bi-manually. This allows us to study how individuals deal with coordination costs in non-social scenarios and compare them to decisions for joint actions. In 50% of trials, one of the participants, henceforth the decision-maker, decided whether to perform the task alone or together. Performing the task alone implied clearing all items presented on the screen and gaining one point for each cleared item. Performing the task together implied splitting the same number of items with the partner (figure 1*d*). Crucially, when performing the task together, participants had to tap on the same item at the same time. Such coordination requirements induce coordination costs in the together condition (figure 1*b*). In Exp. 1, we manipulated action difficulty by introducing trials with a different number of targets, therefore more actions are needed to complete the trial. The primary dependent variable was the proportion of together choices, i.e. the proportion of trials where decision-makers chose to perform a joint action. The second dependent variable was the average trial time, i.e. the time to complete a single trial (Exp. 1, 2 and 3), and the average movement time, defined as the time to perform a tapping action (Exp. 4 and 5). In the electronic supplementary material, we report analyses on number of errors, i.e. the number of extra attempts required to clear a target; asynchrony, i.e. the time interval between partners’ tapping actions in trials requiring coordination; distance travelled (Exp. 4 and 5), i.e. the average two-dimensional trajectory of tapping movements in all conditions.

Exp. 2 provided a solo control for Exp. 1. The aim was to test if individuals (i) were sensitive to maximizing their utility in a solo task, i.e. they were sensitive to the cost–benefit manipulation we introduced, and (ii) showed a preference for action coordination in a non-social scenario, (iii) show different decision patterns in the face of individual coordination costs versus interpersonal coordination costs (Exp. 1). Participants performed the task alone and choose between completing trials either uni-manually (tapping with one hand) or bi-manually (coordinating tapping movements with their two hands). As there is overlap of motor processes regulating intra- and interpersonal hand and limb coordination [48], the uni-manual/bi-manual task modes in this experiment mirror the alone/together modes in Exp. 1 from an action coordination standpoint.

In Exp. 3, we tested the preference for joint action while stressing the importance of maximizing the score. We modified the task so that participants had to reach a target score to finish the experiment: the best task strategy would again be to choose the task mode maximizing points per trial. We also reduced the targets' size in half of the trials. As tapping is more difficult on smaller targets [49], this manipulation introduced higher action difficulty.

In Exp. 4, we tested the preference for joint action in the face of higher coordination difficulty. We modified temporal coordination constraints by introducing Hard and Easy coordination trials that provided individuals with a comparative experience of different coordination constraints. We increased spatial coordination constraints by randomizing location and distance between targets at every trial. Finally, we introduced a ‘time out’ so that every trial had a limited duration (5 s). This created additional pressure to perform each movement fast and accurately and underlined the relationship between actions costs incurred at each trial and the overall success of the task.

Exp. 5 provided a solo control for Exp. 4. Participants performed the task alone and were asked to choose between completing trials either uni-manually (tapping with one hand) or bi-manually (coordinating their tapping movements with their two hands).

## 2. Results

### (a) Experiment 1

**Figure 2.**
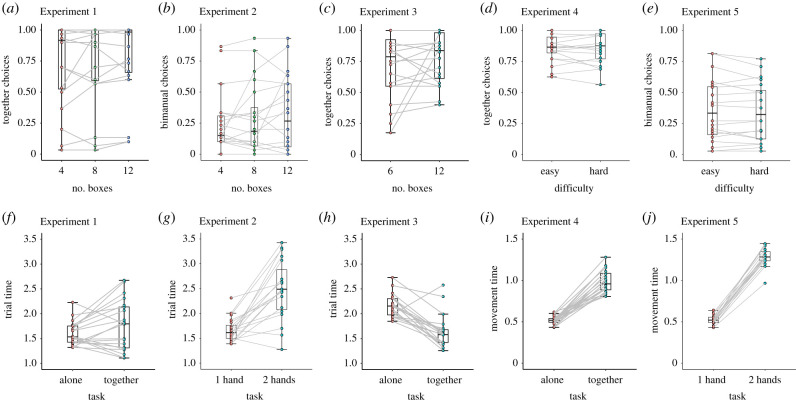
(*a–e*) Boxplots of proportions of together choices in Exp. 1, 3 and 4 and bi-manual choices in Exp. 2 and 5. (*f– j*) Boxplots of within-subjects average trial time in Exp. 1, 2 and 3 and movement time in Exp. 4 and 5, expressed in seconds. Scatter plots connected with grey lines indicate mean values of individual participants in each condition (within subjects). Boxplots and whiskers do not include outliers. (Online version in colour.)

The average proportion of together choices (*M* = 0.76, s.d. = 0.323) was significantly larger than chance level (*V* = 184, *p* = 0.003, *r* = 0.752, CI = 0.44, 0.9). This indicates that participants preferred to perform the task together more frequently than alone (figure 2*a*). The results of the repeated measures analysis of variance (rANOVA) on trial time (Greenhouse–Geisser corrected) showed a significant main effect of task (*F*_1,19_ = 5.521, *p* = 0.03, $\eta_{p}^{2}=0.22$), where participants were faster at performing the task alone (*M* = 1.58 s, s.d. = 0.22) compared to together (*M* = 1.80 s, s.d. = 0.48) (figure 2*d*). The rANOVA also showed a significant main effect of number of boxes (*F*_1,19_ = 472.16, *p* < 0.001, $\eta_{p}^{2}=0.96$), indicating trial duration was longer the more actions participants performed to complete the trial. The significant task × number of boxes interaction (*F*_1,19_ = 4.19, *p* = 0.039, $\eta_{p}^{2}=0.181$) shows that participants were significantly faster alone than together in 12 boxes trials (*p* = 0.017, Bonferroni corrected), while for four and eight boxes trials the difference was not significant (*p*s > 0.1).

### (b) Experiment 2

The average proportion of bi-manual choices (*M* = 0.284, s.d. = 0.259) was significantly smaller than chance level (*V* = 28, *p* = 0.004, *r* = −0.634, CI = −0.859, −0.204). This indicates that participants chose to perform the task uni-manually more frequently than bi-manually (figure 2*b*). The results of the rANOVA on trial time (Greenhouse–Geisser corrected) show a significant main effect of task (*F*_1,19_ = 23.724, *p* < 0.001), $\eta_{p}^{2}=0.557$), where participants were faster at performing the task uni-manually (*M* = 1.66 s, CI = 1.390, 1.927) compared to bi-manually (*M* = 2.55 s, CI = 2.279, 2.287) (figure 2*e*). The rANOVA also revealed a significant main effect of number of boxes (*F*_1,19_ = 279.31, *p* < 0.001, $\eta_{p}^{2}=0.936$), where trial duration was larger the more actions participants performed to complete the trial. The significant task × number of boxes interaction (*F*_1,19_ = 13.87, *p* < 0.001, $\eta_{p}^{2}=0.422$) shows that participants were significantly faster uni-manually than bi-manually in eight boxes trials (*p* = 0.003) and 12 boxes trials (*p* < 0.001), while in four boxes trials *p* > 0.1.

### (c) Experiment 3

The average proportion of together choices (*M* = 0.745, s.d. = 0.205) was significantly larger than chance level (*V* = 198, *p* < 0.001, *r* = 0.890, CI = 0.728, 0.958). This indicates that participants preferred to perform the task together more frequently than alone (figure 2*c*).

The results of the rANOVA on trial time show a significant main effect of task (*F*_1,19_ = 35.7, *p* < 0.001, $\eta_{p}^{2}=0.653$), where participants were faster at performing the task together (*M* = 1.75 s, s.d. = 0.45) compared to alone (*M* = 2.1, s.d. = 0.27) (figure 2*f*). There was also a significant main effect of number of boxes (*F*_1,19_ = 1971.14, *p* < 0.001, $\eta_{p}^{2}=0.990$), where trial duration was longer the more actions participants performed to complete the trial, and a significant main effect of boxes size (*F*_1,19_ = 219.37, *p* < 0.001, $\eta_{p}^{2}=0.92$), where participants were faster at completing the trial when boxes were bigger (as predicted by Fitts law). All other *p-*values > 0.137.

### (d) Experiment 4

The average proportion of together choices (*M* = 0.862, s.d. = 0.118) was significantly larger than chance level (*V* = 210, *p* < 0.001, *r* = 1.000, CI = 1.000, 1.000). This indicates that participants preferred to perform the task together more frequently than alone (figure 2*a*). When testing the average proportion of together choices in easy and hard trials with a paired-sample *t*-test, we found no difference (*V* = 68, *p* = 1.000, *r* = 0.000, CI = −0.506, 0.500). The average proportion of together choices in easy and hard trials depending on the number of boxes was not significantly different.

The results of the rANOVA on movement time show a significant main effect of task (*F*_1,19_ = 225.3, *p* < 0.001, $\eta_{p}^{2}=0.922$), where participants were slower at performing the task together (*M* = 0.988 s, s.d. = 0.136) compared to alone (*M* = 0.517, s.d. = 0.51) (figure 3*c*). There was also a significant main effect of number of boxes (*F*_1,19_ = 27.5, *p* < 0.001, $\eta_{p}^{2}=0.591$), where trial duration was longer the more actions participants performed to complete the trial, and a significant main effect of coordination difficulty (*F*_1,19_ = 72.13, *p* < 0.001, $\eta_{p}^{2}=0.792$), where participants were faster in easy trials (*M* = 0.691 s, s.d. = 0.52) compared to hard trials (*M* = 0.815 s, s.d. = 0.104). There was a significant task × difficulty interaction (*F*_1,19_ = 64.63, *p* < 0.001, $\eta_{p}^{2}=0.773$), indicating that there was significant difference between easy and hard trials only in the together condition (*p* < 0.001). Also the task × number of boxes interaction was significant (*F*_1,19_ = 6.75, *p* = 0.018, $\eta_{p}^{2}=0.262$), indicating that participants when performing the task alone were faster in four boxes trials compared to eight boxes trials (*p* < 0.001). All other *p*s > 0.056.

### (e) Experiment 5

The average proportion of bi-manual choices (*M* = 0.349, s.d. = 0.247) was significantly smaller than chance level (*V* = 42, *p* = 0.02, *r* = −0.6, CI = −0.832, −0.191). This indicates that participants chose to perform the task uni-manually more frequently than bi-manually (Figure 3*b*). When testing the average proportion of bi-manual choices in easy and hard trials with a paired-sample *t*-test, we found no difference (*V* = 115, *p* = 0.43, *r* = 0.211, CI = −0.290, 0.621). When testing the average proportion of bi-manual choices in easy and hard trials depending on the number of boxes with a paired-sample *t*-test, we found that participants chose to perform the task bi-manually in hard, eight boxes trials less frequently compared to all other conditions (all *p* < 0.016).

The results of the rANOVA on movement time show a significant main effect of task (*F*_1,19_ = 463.203, *p* < 0.001), $\eta_{p}^{2}=0.961$), where participants were faster at performing the task uni-manually (*M* = 0.525 s, CI = 0.431, 0.640) compared to bi-manually (*M* = 1.35 s, CI = 0.966, 1.94) (figure 3*d*). The rANOVA also revealed a significant main effect of number of boxes (*F*_1,19_ = 41.06, *p* < 0.001, $\eta_{p}^{2}=0.684$), where trial duration was larger the more actions participants performed to complete the trial. The significant task × coordination difficulty interaction (*F*_1,19_ = 25.41, *p* < 0.001, $\eta_{p}^{2}=0.572$) shows that participants were significantly faster uni-manually than bi-manually in hard trials (*p* = 0.003).

See electronic supplementary material for more analyses.

## 3. Discussion

We investigated how human adults decide when performing a task together with a partner or alone. While it is straightforward to explain a decision to act together when it allows us to achieve outcomes that could not otherwise be achieved, the puzzling scenarios are those where cooperation comes with a direct cost for the individual. Such cases pose a challenge for social cognition theories that model agents' decisions in social scenarios.

Our results indicate that human adults, when faced with the choice of performing a task together or alone, show a preference for joint actions (Exp. 1, 3 and 4). This is the case despite the costs that coordination adds to the task, as demonstrated by the significant performance differences between alone and together condition. In the present task, the costs of coordinating with a partner—synchronizing in space and time and predicting the partner's actions—resulted in observable behavioural costs, i.e. the time, number of actions and amount of movement required to complete together trials. Similar behavioural costs, when experienced in a solo task that agents could perform either uni-manually or bi-manually (Exp. 2 and 5), led individuals to prefer to solve the task uni-manually to minimize their costs. When participants were explicitly instructed to maximize their score, they still preferred to complete the task together, although this choice did not maximize their chance to score points (Exp. 3). Coordination cost in Exp. 3 was reduced compared to Exp. 1, implying that participants performed joint action more efficiently. Performing the task together was still suboptimal, as participants were not fast enough in together trials to guarantee the same number of points compared to alone. In Exp. 4, participants performed the task under conditions of higher coordination difficulty and they were pressured to perform the task fast and accurately. The findings replicate a preference for joint action even when participants were significantly slower and less accurate due to additional coordination costs (hard coordination trials). The analysis of the number of errors confirms participants were less accurate in together/bi-manual trials, and errors increased as a function of task difficulty (electronic supplementary material, Analysis 4). The analysis on distance travelled in Exp. 4 and 5—the two-dimensional trajectory performed at each trial from the first to the last box position—indicates that individuals covered more distance when performing actions in the coordination conditions (together–bi-manual) compared to when acting alone (electronic supplementary material, Analysis 5). Finally, our supplementary analyses on choices over time show that individual preferences did not change over the course of the experiments (while performance level did, see electronic supplementary material, Analysis 2). The results from our five experiments provide consistent evidence that individuals preferred to act together rather than alone although it was not the best available action alternative. When performing the task alone, individuals chose the action alternative that minimized their action costs [22,24,25,50,51].

As the task is designed so that the together action alternative is always instrumentally disadvantageous in terms of how many points (reward) can be gained at each trial, the together/bi-manual task model is suboptimal. The best strategy to solve this task is to choose the action mode that allows the maximum number of points to be collected per trial, and this was indeed the strategy adopted in the solo experiments (Exp. 2 and 5). A utility model that only considers the instrumental costs and rewards of actions is not enough to explain why participants instead engaged in a joint action with a partner when a better (less costly) action alternative was available.

If we only considered the instrumental features of the action alternatives (time, number of actions and points), we would expect opposite patterns of decisions to those observed in Exp. 1, 3 and 4. Our findings reveal that other factors—the expected costs and rewards of acting together—played a role in the decision for joint action and therefore should be accommodated within the utility model participants may have applied at the task. Alternatively, our findings may indicate that participants' decisions for joint action are independent of any cost–benefit analysis and are guided by a bias to engage in cooperation at all costs and independently of outcome. Although we cannot rule out this explanation entirely, we believe it is unlikely that individuals evaluate cooperation opportunities independently of their actual or expected outcome, given the importance of cooperation success in human social life and in the process of partner selection.

This leaves open the question of whether other biases might affect the computation of the utility of acting together. For example, that (i) cooperation always has a higher pay-off than individual action, (ii) outcomes achieved through cooperation are more valuable or (iii) when cooperating the efforts are always shared. It is also possible that, in our studies, participants engaged more frequently in joint actions because they enjoyed acting together more than acting alone without any strategic consideration. This possibility would directly point at a ‘purely rewarding’ nature of performing actions together.

Importantly, there are some limitations to the generalizability of our results. Despite that participants were instructed not to communicate during the experiment, they acted on the apparatus next to each other. The mere presence of a partner in the room may have therefore influenced their decision to engage in joint actions. A pressure to engage in joint action could also derive from the need to manage their reputation with the cooperative partner [52]. Although we instructed decision-makers about the role of their partners (helpers did not collect points and were monetary rewarded independently of their involvement in the task), we acknowledge that reputation management could have affected participants decisions. Further research is needed to explore the influence of physical proximity and reputation on the preference for joint actions. Even when recognizing the influence of such factors, the decision to cooperate on this task prevails over more instrumental motives, in stark contrast with the mutualistic (only) cooperative choices observed in all other apes [53]. We speculate that the preference for cooperation possibly originates in a higher reward value assigned by individuals to acting together, as they may hold expectations of benefits from cooperation beyond its instrumental utility, or they might find acting together more enjoyable without strategic motive. With the current findings, we cannot disentangle whether the value originates from a utility calculus that incorporates not only instrumental but also social benefits and social costs, or it is a generic added value assigned to acting together.

We advance the proposal that a preference for joint action can be understood as an investment in learning to cooperate. One important consideration is that such preference—either originating from a bias to act together independently of the costs and outcomes, or by downplaying the costs/overestimating the benefits of cooperation—incentivizes individuals to engage in cooperation more frequently than they would otherwise, especially when cooperation does not provide immediate instrumental benefit. Frequent engagement in cooperation from early on in life would provide unique learning opportunities for acquiring skills that are crucial for individuals' fitness. Second, it would provide learning opportunities about partners’ cooperative skills and dispositional properties (such as trustworthiness and commitment), which could inform future partner choice [47]. This would reconcile with findings from developmental science showing that children choose cooperation even when is not necessary [6]. The present approach can be applied to address a variety of open questions about human preferences for joint action and allows predictions to be formulated based on the value assigned to cooperative actions. One outstanding question is whether there is a tipping point in coordination costs that would shift participants' preference towards individual goal achievement. A further important question is whether preference for joint action is modulated by the kinds of coordination cost: likelihood of task failure, error monitoring, cognitive load are just some candidate factors that may be crucial in deciding whether to engage in joint action or not. Another outstanding question is whether participants’ preference would change as a function of the interaction history with a partner.

In conclusion, we propose a framework to systematically investigate adults' preference for cooperation through joint action. It allows us to pinpoint the types and the extent of costs that human adults—and potentially other species—accept to engage in cooperation with partners. It has also the potential of identifying cultural and individual differences in integrating coordination costs in the decision for joint action.

## 4. Methods

### (a) Experiment 1

#### (i) Task

Participants cleared boxes by tapping with their finger on the boxes’ surfaces in any order they preferred. A trial ended when all boxes were cleared. In 50% of the trials, one of the participants, henceforth the decision-maker, decided whether to perform the task *alone* or *together*. To clear the boxes, both participants had to tap on the boxes with the same position on the grid, and at the same time, within a pre-specified time window (200 ms). If the taps were not synchronous or did not land on the same box, the box remained on the screen and had to be tapped again.

#### (ii) Participants

Exp. 1, 2 and 3 in this study were approved by the United Ethical Review Committee for Research in Psychology (EPKEB). All participants signed prior informed consent and received monetary compensation that was independent of their task performance. The study was performed in accordance with the Declaration of Helsinki and later amendments. We recruited 20 pairs in total (40 participants) (15 F; average age = 26.06 ± 4.47 years).

#### (iii) Apparatus and stimuli

The task was performed on an Iiyama 46″ PROLITE TF4637MSC-B2AG touchscreen set (1600 × 900 pixels resolution). The screen area was divided vertically into two equal halves, corresponding to the participants' workspace. Coloured squares (boxes) were displayed in a grid arrangement, equidistant from each other. Boxes were blue during the trial (when they were active) and green during preview (see Procedure). The experimental script, trial randomization and participants’ responses were controlled using MatLab 18b software.

#### (iv) Procedure

Participants came into the testing room in pairs and stood in front of a touchscreen that was lying flat on a table ( figure 1*c*). The roles of decision-maker and partner were randomly assigned. As no hypotheses about the effect of gender on the current research questions were formulated, pairs of participants were randomly composed without controlling for gender. The task for the decision-maker was to clear as many boxes as she could within 25 min. Unbeknownst to the participants, the number of trials was fixed, and the time limit was set for longer than the time required to finish the task on average. To highlight the relevance of time, we installed an electronic countdown clock on the desk in front of the participants.

The screen side (left–right) occupied by the decision-maker was counterbalanced across participants. Participants received no specific instructions about which finger to use to clear boxes, but from piloting, we observed that everyone used the index finger of their dominant hand. We ensured that participants could comfortably move their hands without spatial constraints, regardless of what hand they used to complete the task. We presented three trial types in random order: no-choice alone, no-choice together and choice trials. At each trial four, eight or 12 boxes were displayed on the screen (see electronic supplementary material, table S1 for trial counts). We administered a fixed number of no-choice together and no-choice alone trials to ensure that each participant experienced the same number of trials in each task mode, independently of her decisions in choice trials. The order of conditions was fully randomized. Before proceeding to the experiment, participants received four practice trials to familiarize themselves with the procedure. At the beginning of each trial, the decision-maker watched a preview of the upcoming trial configuration followed by a display of two buttons (alone and together). By pressing one, the decision-maker started the trial in the mode she selected and boxes changed colour to signal they were active. One of the buttons was inactive in the no-choice trials but visually identical. During preview, a text box displayed the total number of boxes cleared so far (decision-maker's score). In alone trials, boxes appeared only on the decision-maker's side of the screen. In together trials, half of the boxes appeared on the decision-maker's side and half on her partner's side of the screen. In this condition, boxes on the two halves of the screen had to be cleared by the two participants, by touching synchronously on them. By choosing together, decision-makers scored points only for the boxes cleared on their side of the screen. No time constraint was imposed on trial completion. Participants were instructed not to communicate with each other, verbally or otherwise, and to look at the touchscreen in front of them.

#### (v) Data analyses

The primary dependent variable was the proportion of together choices, that is, the proportion of trials where decision-makers chose to perform a joint action. To test whether individuals had a preference for together trials over alone trials, we tested participants' average choices against chance level. The second dependent variable was the average trial time for no-choice trials, defined as the time to complete the trial calculated from the first touch to the touch that cleared the last box on the screen. Trials that were above or below 3 standard deviations from the sample mean, calculated across conditions for each level separately, were excluded from the analysis (1.5% of all trials). No participants were excluded. We performed a 2 × 3 rANOVA with task (alone, together) and number of boxes (4, 8, 12) as within-subject factors. See electronic supplementary material for assumption checks and summary statistics. Data were analysed in JASP v. 0.16 and R Studio 4.1.1.

### (b) Experiment 2

#### (i) Participants

We recruited 20 participants in total (8 F; average age = 25.8 ± 5.17 years).

#### (ii) Apparatus and stimuli

The same apparatus and stimuli of Exp. 1.

#### (iii) Procedure

Single participants (decision-maker) choose between two buttons (one hand and two hands). In uni-manual (one hand) trials, boxes appeared only on one side of the screen (counterbalanced across participants) and could be cleared with a single tap on each item. In bi-manual (two hands) trials, half of the boxes appeared in front of the decision-maker and the other half appeared on the other side of the screen. In this condition, boxes on the two halves of the screen had to be cleared simultaneously by means of a synchronous touch on the two corresponding boxes with her two hands. The participant could comfortably reach both sides of the screen. By choosing ‘two hands’, decision-makers scored points only for the boxes they cleared on one side of the screen. The procedure is otherwise the same as Exp. 1.

#### (iv) Data analyses

Data analyses and trial exclusions is the same as Exp. 1 (exceeding +/− 3 s.d.; 1.5% of the total trial number). One participant was excluded as an outlier.

### (c) Experiment 3

#### (i) Participants

We recruited 20 pairs in total (36 F; average age = 23.13 ± 2.95 years).

#### (ii) Apparatus and stimuli

The experiment was conducted on a 43″ Iiyama PROLITE TF4338MSC-B1AG touchscreen. For more details see electronic supplementary material.

#### (iii) Procedure

Participants were instructed that their goal was to reach the target score of 1500 points. Unbeknownst to the participants, the number of trials was fixed and the target score was larger than the score they would reach by finishing the trials. In together trials, participants had to tap on the boxes with the same position on the grid within a pre-specified time window of 250 ms. In addition to trial type (no-choice alone, no-choice together and choice) and number of boxes (six and 12), there was a third factor of boxes size with two levels: big and small (half the size of big) boxes.

#### (iv) Data analyses

Data analyses and trial exclusions is the same as Exp. 1 (exceeding +/− 3 s.d.; 2.6% of the total trial number). For more details see electronic supplementary material.

### (d) Experiment 4

#### (i) Participants

We recruited 20 pairs in total (27 F; average age = 26.2 ± 4.6 years). Exp. 4 and 5 in this study were approved by the Psychological Research Ethics Board (PREBO, reference number 2021_10).

#### (ii) Apparatus and stimuli

The apparatus was identical to previous experiments. For more details see electronic supplementary material.

#### (iii) Procedure

Participants were instructed that each trial had a fixed duration (5 s) and their task was to clear as many boxes as possible. When trial time was over, all targets still on screen would disappear and the points would be lost. Trial time was displayed on the touchscreen (figure 1*c*). In addition to trial type (no-choice alone, no-choice together and choice) and number of boxes (4 and 8), there was a third factor of coordination difficulty with two levels: easy and hard trials. In together easy trials, participants had to tap on the boxes with the same position on the grid within a pre-specified time-window of 120 ms, while in hard trials the time window was 60 ms.

Procedure is otherwise the same as Exp. 1. The experimental script, trial randomization and participants' responses were controlled with a Max MSP patch. For more details see electronic supplementary material.

#### (iv) Data analyses

Data analyses and trial exclusions were the same as other experiments (exceeding +/− 3 s.d.; 1.04% of the total trial number). For more details see electronic supplementary material.

### (e) Experiment 5

#### (i) Participants

We recruited 20 participants in total (12 F; average age = 26.65 ± 7.17 years).

#### (ii) Apparatus and stimuli

Same apparatus and stimuli of Exp. 4. The experimental script, trial randomization and participants’ responses were controlled using a Max MSP patch.

#### (iii) Procedure

Single participants (decision-maker) choose between two buttons (one hand and two hands). The participant could comfortably reach both sides of the screen. By choosing ‘two hands’, decision-makers scored points only for the boxes they cleared on one side of the screen. The procedure is otherwise the same as Exp. 4.

#### (iv) Data analyses

Data analyses and trial exclusions is the same as Exp. 1 (exceeding +/− 3 s.d.; 1.15% of the total trial number). For more details see electronic supplementary material.

The design, procedure and analysis plan for Exp. 4 and 5 were pre-registered at: https://aspredicted.org/jy6bb.pdf.

The electronic supplementary material is available online. Data and analyses scripts are available in the following repository: https://doi.org/10.5061/dryad.nvx0k6dv2.

## Data Availability

All data are available from the Dryad Digital Repository: https://doi.org/10.5061/dryad.nvx0k6dv2 [54].

## Endnote

1 We use cooperation/cooperative activities to refer to behaviours providing a benefit to another individual (recipient) or are beneficial to both the actor and the recipient [8]. We use joint action to refer to the coordination of instrumental actions in space and time to bring about a change in the environment, [7] which is a prerequisite for some forms of cooperation.
